# The Efficacy and Potential Mechanisms of Metformin in the Treatment of COVID-19 in the Diabetics: A Systematic Review

**DOI:** 10.3389/fendo.2021.645194

**Published:** 2021-03-19

**Authors:** Moein Zangiabadian, Seyed Aria Nejadghaderi, Mohammad Mahdi Zahmatkesh, Bahareh Hajikhani, Mehdi Mirsaeidi, Mohammad Javad Nasiri

**Affiliations:** ^1^ Student Research Committee, School of Medicine, Shahid Beheshti University of Medical Sciences, Tehran, Iran; ^2^ Department of Microbiology, School of Medicine, Shahid Beheshti University of Medical Sciences, Tehran, Iran; ^3^ Department of Medicine, Division of Pulmonary, Critical Care, Sleep and Allergy, University of Miami, Coral Gables, FL, United States

**Keywords:** metformin, diabetes mellitus, SARS-CoV-2, COVID-19, 2019-nCoV, systematic review

## Abstract

**Introduction:**

Diabetes mellitus (DM) is one of the most common comorbidities among patients with coronavirus disease 2019 (COVID-19) which may exacerbate complications of this new viral infection. Metformin is an anti-hyperglycemic agent with host-directed immune-modulatory effects, which relieve exaggerated inflammation and reduce lung tissue damage. The current systematic review aimed to summarize the available evidence on the potential mechanism of action and the efficacy of metformin in COVID-19 patients with DM.

**Methods:**

A systematic search was carried out in PubMed/Medline, EMBASE, the Cochrane Controlled Register of Trials (CENTRAL), and Web of Science up to July 30, 2020. The following keywords were used: “COVID-19”, “SARS-CoV-2”, “2019-nCoV”, “metformin”, and “antidiabetic drug”.

**Results:**

Fourteen studies were included in our systematic review. Three of them were observational with 6,659 participants. Decreasing insulin resistance, reduction of some inflammatory cytokines like IL-6 and TNF-α, modulation of angiotensin-converting enzyme 2 (ACE2) receptor, and improving neutrophil to lymphocyte ratio are some of the potential mechanisms of metformin in COVID-19 patients with DM. Nine out of fourteen articles revealed the positive effect of metformin on the prognosis of COVID-19 in diabetic or even non-diabetic patients. Moreover, different studies have shown that metformin is more effective in women than men.

**Conclusions:**

The use of metformin may lead to improve the clinical outcomes of patients with mild to moderate SARS-CoV-2, especially in diabetic women. Further observational studies should be conducted to clarify the effects of metformin as a part of the treatment strategy of COVID-19.

## Introduction

In late December 2019, a cluster of patients with pneumonia was referred to hospitals in Wuhan city, Hubei province, China (1). Further clinical investigations showed that a novel coronavirus called Severe Acute Respiratory Syndrome Coronavirus-2 (SARS-CoV-2) caused the disease, COVID-19 (2). Rapidly, COVID-19 affected almost all countries and territories worldwide, so that the World Health Organization (WHO) declared it as a pandemic on March 11, 2020 (3). As of late February 2021, more than 112 million prevalent numbers and above 2.5 million deaths were due to affecting by SARS-CoV-2 (4). Some pharmacologic therapeutic approaches like chloroquine, lopinavir/ritonavir, favipiravir, and tocilizumab have been used in clinical practice without any proven effect (5), while some drugs such as remdesivir have shown some effects on reducing time to recovery (6). Seniors, patients with preexisting diseases including hypertension, diabetes, coronary heart disease, and chronic obstructive pulmonary disease are at a higher risk of morbidity and mortality of COVID-19 (7).

Based on findings of a study on diabetic patients compared to controls, inflammatory responses and levels of inflammation-related biomarkers are higher among patients with diabetes mellitus (DM), which suggests considering DM as a major risk factor in the progression and prognosis of SARS-CoV-2 (8). DM is one of the most common comorbidities among COVID-19 patients, which leads to the intensive care unit (ICU) admission in 14%-32% of cases (9). Different potential mechanisms were mentioned for severity and increased risk of COVID-19 in patients with DM, including upregulation of angiotensin-converting-enzyme-2 (ACE2) expression (10), increased interleukin-6 (IL-6) expression (11), and decreasing CD4-positive T-cells in diabetic patients with Mediterranean Eastern respiratory syndrome coronavirus (MERS-CoV) (12) that may have the similar mechanism with COVID-19.

Metformin is a well-known antidiabetic drug, which showed immunomodulatory effects by phosphorylation of adenosine monophosphate (AMP)-activated protein kinase in animal models (13). A large-scale observational study showed that metformin reduced the mortality of chronic lower respiratory diseases significantly compared to the overall population (14).

This systematic review aimed to determine the potential efficacy of metformin in COVID-19 and the underlying mechanism of action in patients with preexisting DM.

## Methods

This systematic review was according to the “Preferred Reporting Items for Systematic Reviews and Meta-analyses” (PRISMA) statement (15).

### Search Strategy

A systematic search was carried out in the literature from the following bibliographical databases: PubMed/Medline, EMBASE, the Cochrane Controlled Register of Trials (CENTRAL), and Web of Science up to July 30, 2020. The following search terms were used: “COVID-19”, “SARS-CoV-2”, “2019-nCoV”, “metformin”, and “antidiabetic drug”. Lists of references of selected articles and relevant review articles were hand-searched to identify further studies. There were no restrictions on publication date and type of article but only studies written in English were selected.

### Study Selection

All potentially English relevant articles were screened in two stages for eligibility. In the first stage, two reviewers independently reviewed titles and abstracts and chose those fitting selection criteria for full-text evaluation. Discrepancies were discussed with a third reviewer. In the second stage of assessment with full-text evaluation, all types of studies except reviews that discussed the effect of metformin in different stages of the COVID-19 and represented its mechanism were considered by the same authors. Disagreements and technical uncertainties were discussed and resolved between review authors.

### Data Extraction

The following variables were extracted from all included studies: first author, year of publication, type of study, country/ies where the study was conducted, viral entrance pathway of SARS-CoV-2, mechanism of action of metformin against COVID-19, other effective drugs with the role of them, Possibility of using metformin in COVID-19 treatment and target society. In three analytic studies the extracted data included: study population (number of cases with metformin and number of controls without metformin), mean age (both in metformin and no-metformin group), COVID-19 diagnosis technique (clinical and confirmed diagnosis), and the effect of metformin on outcomes (effect on the duration of hospitalization, the effect on in-hospital death, and effect on poor prognosis). Two authors independently extracted the data from the selected studies. The data was jointly reconciled, and disagreements were discussed and resolved between review authors.

## Results

**Figure 1:**
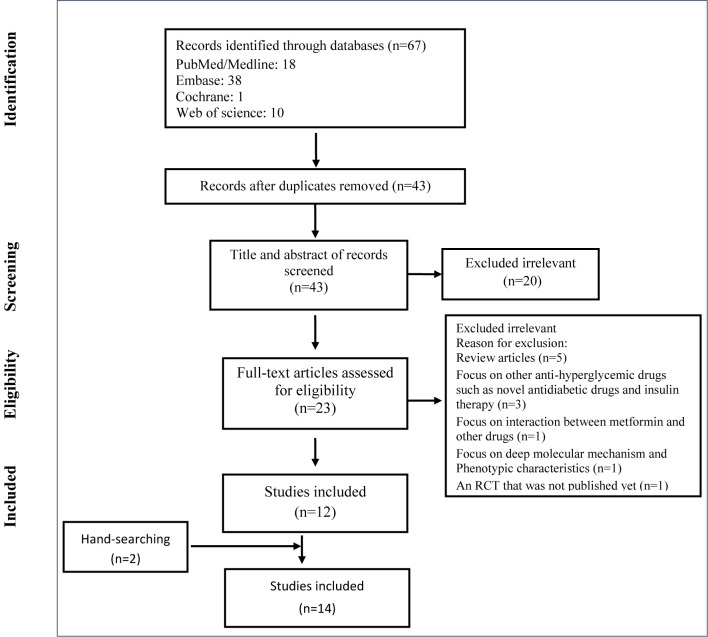
Flow chart of study selection for inclusion in the systematic review.

The selection process of articles is shown in **Figure 1**. Fourteen articles were included and classified into the followings: three commentaries (16–18), six letters to the editor (19–24), three observational studies (25–27), one personal view (28), and one research prospective study (29) (**Table 1**). Two of the three observational studies were conducted in China and one in the USA. The total population of the observational articles was 6,659. History of exposure, clinical symptoms, imaging findings, and laboratory assessments was included in clinical diagnosis. Also, PCR or immunoglobulin (Ig)-M and IgG tests were used to confirm the diagnosis (**Tables 1** and **2**).

**Table 1:** Characteristics of all included studies.

Title of article	Author	Year	Country	Type of study
Metformin, neutrophils and COVID-19 infection	Dalan et al. (16)	2020	Singapore	Commentary
Metformin in COVID-19: A possible role beyond diabetes	Sharma et al. (17)	2020	India	Commentary
Is metformin ahead in the race as a repurposed host-directed therapy for atients with diabetes and COVID-19?	Singh et al. (9)	2020	India	Commentary
COVID-19 and Diabetes Mellitus: May Old Antidiabetic Agents Become the New Philosopher’s Stone?	Penlioglu et al. (23)	2020	Greece	Letter to the editor
COVID-19 and diabetes: Is metformin a friend or foe?	Ursini et al. (24)	2020	Italy	Letter to the editor
Metformin use amid coronavirus disease 2019 pandemic	Kow et al. (22)	2020	Malaysia and UK	Letter to the editor
Metformin and COVID-19: A novel deal of an old drug	El-arabey et al. (19)	2020	Egypt and China	Letter to the editor
A proposed mechanism for the possible therapeutic potential of Metformin in COVID-19	Esam et al. (21)	2020	Iran	Letter to the editor
Comment on ‘‘Should anti-diabetic medications be reconsidered amid COVID-19 pandemic?’’	Cure et al. (20)	2020	Turkey	Letter to the editor
Metformin Treatment Was Associated with Decreased Mortality in COVID-19 Patients with Diabetes in a Retrospective Analysis	Luo et al. (27)	2020	China	Retrospective analysis
Observational Study of Metformin and Risk of Mortality in Persons Hospitalized with COVID-19	Bramante et al. (25)	2020	USA	Retrospective cohort analysis
Clinical Characteristics and Outcomes of Patients With Diabetes and COVID-19 in Association With Glucose-Lowering Medication	Chen et al. (26)	2020	China	Retrospective analysis
Practical recommendations for the management of diabetes in patients with COVID-19	Bornstein et al. (28)	2020	Switzerland, Germany, UK, Italy, Singapore, USA, Netherlands, France, Australia, Brazil, Spain, China	Personal view
Metformin and SARS-CoV-2: mechanistic lessons on air pollution to weather the cytokine/thrombotic storm in COVID-19	Menendez et al. (29)	2020	Spain	Research perspective

UK, United Kingdom; USA, United States of America; COVID-19: Coronavirus disease 2019; SARS-CoV-2, Severe acute respiratory syndrome coronavirus 2.

**Table 2:** Detailed characteristics and statistical information of three retrospective analytical studies.

Authors	Study population			Age (mean)^4^		COVID-19 diagnosis technique		Outcome		
	Used metformin	Did not use metformin	total	Metformin group	No metformin group	Clinical diagnosis	Confirmed diagnosis	Effect on duration of hospitalization	Effect on in-hospital death	Effect on poor prognosis
Luo et al.	104	179	283	63	65	History and clinical symptoms. Imaging (CT scan) Laboratory assessment	Nuclear acid test or IgM-IgG test	No significant effect^1^	Significantly decreased OR:0.21 (0.06-0.73) P-value:0.02	–
Bramante et al.	2333	3923	6256	73	76	–	PCR	–	Significantly decreased in women^2^ OR:0.792 (0.640-0.979) P-value:0.031	–
Chen et al.	43	77	120	62	67	Clinical symptoms, exposure, imaging findings	NGS, fluorescent RT-PCR	No significant effect^3^	No significant effect OR:0.62 (0.17-2.20) P-value:0.456	No significant effect OR:2.49 (0.92-6.76) P-value:0.074

COVID-19, Coronavirus disease 2019; Ig, immunoglobulin; CT, computed tomography; OR, odds ratio; RT-PCR, real-time polymerase chain reaction; NGS, next generation sequencing.

1) Duration of hospitalization was 21 days in metformin group while it was 19.5 days in no-metformin group.

2) In overall population metformin was not significantly effective. OR: 0.904 (0.782-1.045); P-value: 0.173

3) Duration of hospitalization was 24 days in metformin group while it was 23 days in no-metformin group.

4) The total mean age in studies were 64.2, 74.8 and 65.2 respectively.

### Possibility of Using Metformin in COVID-19 Treatment

Nine out of fourteen articles showed the efficacy of metformin on COVID-19 that not only can be continued in diabetic patients as an anti-hyperglycemic drug but also may be offered in non-diabetics as a part of treatment protocol (17–19, 21, 23, 25–27, 29). Three articles did not provide a definitive opinion on the use of metformin for therapeutic purposes (16, 22, 24), and two articles reported that metformin should not be used in patients with SARS-CoV-2 infection (20, 28) (**Table 3**). Among these articles, Bramante et al. (25) by designing a retrospective cohort analysis study noticed that metformin has sex-specific effects and is useful for diabetic or obese women. Chen et al. (26) reported the positive effect of metformin in the course of the disease, whereas this effect was not statistically significant. On the contrary, Bornstein et al. (28) reported that metformin should not be used in patients with severe symptoms of COVID-19, but it is not contraindicated in patients with mild or moderate symptoms.

**Table 3:** Possibility of using metformin in COVID-19 treatment.

Author	Possibility of using metformin in COVID-19 treatment	suggested for/not suggested for
Dalan et al.	May	Uncertain
Sharma et al.	Yes	Not mentioned
Singh et al.	Yes	Suggested: Patients with diabetes and COVID-19
Penlioglu et al.	Yes	Suggested: Patients with or without diabetes
Ursini et al.	May	Uncertain
Kow et al.	May	Uncertain
El-arabey et al.	Yes	Suggested: Elderly, obese and diabetics
Esam et al.	Yes	Suggested: patients in acute, chronic and recovery phases of COVID-19
Cure et al.	No	Not suggested in diabetics
Luo et al.	Yes	Both diabetics and non-diabetics
Bramante et al.	Yes in women	Diabetic or obese women
Chen et al.	Yes but not strongly recommended	Not mentioned
Bornstein et al.	No (in patients with severe symptoms of COVID-19)	Not Suggested: patients with severe COVID-19 symptoms
Mendez et al.	Yes	Suggested: patients with severe COVID-19

COVID-19, Coronavirus disease 2019.

### Effect of Metformin on Clinical Outcomes

Duration of hospitalization, in-hospital death, and poor prognosis (progression to severe or critical illness) was discussed as major outcomes in three retrospective analytical studies. Luo et al. (27) and Chen et al. (26) did not report any significant effect of metformin on the duration of hospitalization. Furthermore, according to Luo et al. study (27), there was no significant relationship between metformin and poor prognosis. For in-hospital death, Luo et al. (27) represented a significant decrease in the metformin group and Bramante et al. (25) reported this significant effect only in women. On the other hand, the article by Chen et al. (26) did not represent any significant relationship between the administration of metformin and in-hospital mortality reduction. More details on the three mentioned studies are available in **Table 2**.

### Potential Mechanisms of Metformin and Other Drugs Against SARS-CoV-2

The suggested mechanisms of action for metformin within the fourteen included papers can be classified into some major categories. First, El-Arabey et al. (19) and Kow et al. (22) mentioned that weight reduction might have moderate protective effects against SARS-CoV-2, especially in the elderly. Second, adenosine monophosphate-activated protein kinase (AMPK) pathways that can affect the expression of ACE2, the receptor for SARS-CoV-2, is another pathway that can be modulated by metformin (17, 23–25). The articles by Penlioglou et al. (23), Sharma et al. (17), and Bramante et al. (25) mentioned protective roles of metformin by different mechanisms such as a reduction in insulin resistance (23), improving ACE2 stability (17), balancing renin-angiotensin-aldosterone system (17), modulation of ACE2 receptor (25), and controlling blood glucose levels (25). However, Ursini et al. (24) suggested that overexpression of ACE2 as a result of the AMPK pathway can put diabetic patients at higher risk of affecting by SARS-CoV-2. Third, high production of lactate and lactic acidosis due to metformin might be another potential mechanism for the high rate of infection in patients using metformin (20, 22, 28). Fourth, anti-inflammatory effects of metformin can protect patients administrating metformin from SARS-CoV-2 in different ways like inhibition of cytokine storms (18, 27, 29), inhibition of IL-6 crisis (18), and modulation of gut microbiota composition (18). Fifth, reduction of neutrophil count and improving neutrophil to lymphocyte ratio is another mechanism of metformin against COVID-19 in patients with preexisting diabetes mellitus (16, 25). In addition to these main mechanisms, metformin can play roles in protecting from or predisposing diabetic patients to SARS-CoV-2 infection in other various pathways, including reduction of vitamin B12 and immunosuppression (22), inhibition of PI3K/AKT/mTOR pathway (17), reducing thrombosis formation (25), prevention of lung damage and fibrosis (21, 27), interruption of the endocytic cycle due to decreasing acidity of endosomes and lysosomes (21), and reduction of some inflammatory cytokines like IL-6 (18, 25, 26) and TNF-α (25). **Table 4** shows the mechanisms of metformin and other drugs on SARS-CoV-2 infection in diabetic patients.

**Table 4:** Suggested potential mechanisms of action of metformin and other drugs on Severe Acute Respiratory Syndrome-Coronavirus-2 (SARS-CoV-2).

Author	Suggested viral entrance pathway of SARS-CoV-2	Suggested mechanism of action of metformin on COVID-19.	Other effective drugs	Role of other dugs
El-arabey et al.	Not mentioned	Reduction of weight and pneumonia in elderly, obese and diabetic patients.	Not mentioned	Not mentioned
Mendez et al.	Not mentioned	Preventing ROS/CRAC mediated IL-6 release, preventing cytokine and thrombotic-like storms, ameliorate immunometabolism- related inflammation thus alleviating ARDS.	CM4620 might be useful.	CM4620 is a direct inhibitor of CRAC channel.
Singh et al.	Not mentioned	Anti-inflammatory properties: induction of AMPK, anti-oxidant role by altering activity of catalase and superoxide dismutase, altering composition of gut microbiota to reduce inflammation. Collectively combating cytokine storm and preventing excessive rise in IL-6.	Not mentioned	Not mentioned
Penlioglou et al.	Not mentioned	Reduction in insulin resistance in infected subjects by AMPK, reduction in liver fibrosis and also protective role on liver.	Pioglitazone	Anti-inflammatory, improving liver injury
Ursini et al.	ACE2 acts as docking site for SARS-CoV-2	AMPK Increases ACE2 expression and stability thus promoting SARS-CoV-2 infection. Optimal management of glucose levels and the immune-modulation properties of metformin may result on a beneficial effect on patients’ outcome.	Not mentioned	Not mentioned
Kow et al.	Not mentioned	Only modest weight reduction (may not be the main mechanism to protect against, if any, toward mortality from COVID-19), metformin-induced vitamin B12 deficiency may weaken immune system (patients should undergo routine B12 monitoring). Metformin may cause lactic acidosis.	Not mentioned	Not mentioned
Sharma et al.	ACE2 acts as docking site for SARS-CoV-2	AMPK Increases ACE2 expression. AMPK increases ACE2 stability by phosphorylation. This could lead to decreased binding with SARS-CoV-2 due to steric hindrance. Metformin prevents the detrimental sequelae of imbalance RAS by causing activation of ACE2 through AMPK signaling. Inhibition of P13k/AKT/mTOR pathway. Prevention of viral replication and pathogenesis through inhibition of mTOR pathway. Activated AMPK potentiates expression of certain genes with known antiviral properties such as IFNs, OAS2, ISG15, and MX1 while inhibiting inflammatory mediators like TNF-alpha and CCL5. Role of metformin in optimal control of T2DM, for both chronic and transient cases, might help in the treatment of COVID-19.	2-DG	Inhibition of glycosis
Esam et al.	Endocytosis	Metformin increases endosomal and lysosomal pH through acting directly on V-ATPase as proton pumping or acidifier compartment, and eNHEs as proton leaking or alkalizing compartment on the endosomal membrane and subsequently interferes with the endocytic cycle. Metformin is a strong base drug (pKa= 12.4) which might enhance the pH of the acidic vesicles containing viruses. Metformin can reverse established lung fibrosis.	Not mentioned	Not mentioned
Dalan et al.	Not mentioned	Metformin can reduce neutrophil count and neutrophil to lymphocyte ratio in patients with diabetes. Metformin can reduce NETs formation in diabetics and prediabetes patients. Metformin can reduce neutrophil and macrophage infiltration in hyperoxia induced lung injury (described in neonatal rats).	Not mentioned	Not mentioned
Bornstein et al.	ACE2 acts as docking site for SARS-CoV-2	Metformin can cause lactic acidosis.	ACEi and ARB statins	1) Could protect against severe lung injury following infection 2) administration is not recommended but statins should not be discontinued because of the long-term benefits and the potential for tipping the balance toward a cytokine storm by rebound rises in IL-6 and IL-1β if they were to be discontinued.
Luo et al.	Not mentioned	Metformin can modulate immune mechanisms that relieve cytokine storms and exaggerated inflammation to reduce lung tissue damage.	Chinese traditional medicine such as Lianhua Qingwen capsules	Not mentioned
Bramante et al.	ACE2 acts as docking site for SARS-CoV-2	Metformin decreases levels of TNF-α and IL-6, and boosts levels of IL-10, significantly more in females than males. ACE2 receptor modulation (via AMPK) improved neutrophil to lymphocyte ratio, decreased glycemia (via AMPK), mast cell stabilization, decreased thrombosis, and improved endothelial function.	Beta 2 agonists (used in asthmatics)	IL-10 boosting and TNF-α decreasing in asthmatics
Chen et al.	ACE2 acts as docking site for SARS-CoV-2	Metformin can reduce IL-6.	Not mentioned	Not mentioned
Cure et al.	Penetration to ACE2 at low pH	Metformin increases lactate production.	Insulin Dapagliflozin	1) Lowers ADAM-17 activation and thus reduces ACE2 level. Increases NHE activation. Both reducing possibility of virus adhering cell. 2) Lowers lactate levels.

ROS, reactive oxygen species; CRAC, calcium release-activated channel; ARDS, acute respiratory distress syndrome; ACE2, angiotensin-converting enzyme 2; 2-DG, 2-deoxy-D-glucose; RAS, renin-angiotensin-aldosterone system; mTOR, mammalian target of rapamycin; IL, interleukin; IFN, interferon; TNF, tumor necrosis factor; CCL5, chemokine ligand 5; T2DM, type 2 diabetes mellitus; V-ATPase, Vacuolar ATPase; eNHE, endosomal Na+/H+ exchanger; NET, neutrophil extracellular traps; ACEi, angiotensin-converting enzyme inhibitor; ARB, angiotensin receptor blocker; NHE, Na+/H+ exchanger.

## Discussion

In the present work, most of the evaluated studies have shown that the use of metformin in the treatment regimen of diabetic and non-diabetic patients with COVID-19 is beneficial. Only two studies found that metformin should not be used in severe forms of the COVID-19, however, they did not prohibit its use in moderate to mild cases of infection.

COVID-19 is associated with poor prognosis and increased mortality rate in patients with DM. Also, diabetes management in patients suffering from COVID-19 is a great clinical challenge. Most of the studies evaluated in the current systematic review point to the beneficial effect of metformin in the treatment of COVID-19 patients with diabetes. Metformin as an agent of host-directed therapy can modulate immune mechanisms and therefore might prevent progression to acute respiratory distress syndrome (ARDS). Since there are some metabolic similarities between COVID-19 and DM such as hyperglycemia, oxidative stress, and pro-inflammatory cytokines, it is not unreasonable to expect that metformin with effects such as decreasing the levels of inflammatory cytokines IL-6 and TNF-alpha as well as increasing IL-10, an anti-inflammatory cytokine can play a beneficial role in reducing the complications of COVID-19 in patients with DM (30). Metformin induces the formation of M2 macrophages and T-regulatory as well as CD8 memory T cells which in turn minimize the inflammatory reactions (31). However, some studies have suggested not using this medication in the COVID-19 treatment protocol. In fact, in patients suffering from heart failure, respiratory distress, sepsis or renal impairment use of metformin should be stopped due to the risk of lactic acidosis (28). Of course, it should be noted that the risk of acidosis following the use of metformin is not very high, however, it should be considered, especially in hospitalized patients. Another issue to be considered is that metformin reduces intestinal absorption of vitamin B12 and lowers serum vitamin B12 concentrations in some metformin-treated patients. Due to the role of vitamin B12 in regulating the immune system its deficiency may negatively affect the cellular immune responses and thereafter facilitates infection from COVID-19 (32, 33). So, routine monitoring of vitamin B12 is suggested in metformin-treated patients.

Ursini et al. speculated that metformin synergistically with ACEi or ARBs may theoretically result in increasing ACE2 availability in the respiratory tract thus promoting Coivid-19 (24).

Nevertheless, it has been shown that poorly controlled blood glucose levels make patients prone to experience more complications or in-hospital death due to COVID-19. In other words, hospitalized patients with COVID-19 and diabetes had a longer length of stay in the hospital than patients without diabetes (34). Metformin by enhancing the activity of existing insulin and reducing hepatic glucose production exerts its glucose-lowering effect. That’s why it does not generally cause hypoglycemia in patients with or without diabetes (27). However, since the effects of host-directed therapy agents such as metformin on the virus itself are limited, we do not expect a reduction in the hospitalization period and the time it takes for the patient to be free of the virus (27). Luo et al. (27) and Chen et al. (26) findings are consistent with this theory. However, it has been proposed that metformin may have an inhibitory effect on the virus, by increasing insulin sensitivity (35).

The next issue is the more effective effects of metformin in women compared to men (25, 36). Also, metformin causes a greater reduction in TNF-α and IL-6 in women (36–38). The article by Mackey et al. suggests that this might be due to a higher secretion of TNF-α from mast cells in response to inflammation in women (39). Moreover, Klein et al. claim that sex hormones and epigenetic changes of the Y chromosome may be responsible for the sex-specific effects of metformin (40). Also, Li et al. proposed that although metformin increases the expression of ACE2 in both sexes equally, the subsequent inflammatory response can be different between men and women, which can also have a relation to the sex-specific benefits of metformin (41). Last, of all, metformin can increase the levels of IL-10, an anti-inflammatory cytokine, in women more than men (42, 43). According to the above, it is proven that Metformin is more beneficial in women with DM and COVID-19 compared to similar men.

The beneficial effects of metformin to reduce the duration of hospitalization, in-hospital death and poor prognosis are controversial. Since the studies performed in these cases are limited, evidence may not be sufficient to confirm or deny the adequacy of metformin in reducing these complications in patients with diabetes suffering from COVID-19.

The current study has been limited with a lack of clinical trials and the number of cohort studies. So we couldn’t design a meta-analysis study. Furthermore, only the efficacy of metformin is discussed in our study, and the effects, mechanisms, complications, and interactions of other diabetic and non-diabetic drugs that may use in combination therapy in diabetic patients have not been studied.

## Conclusions

According to the current knowledge, it can be concluded that the use of metformin can have beneficial effects on COVID-19, especially in diabetic patients, while more studies such as retrospective analysis of COVID-19 diabetic cohorts are suggested to be conducted. These beneficial effects of metformin should be considered, especially among female patients. On the other side, in the case of hospitalized patients with severe symptoms of COVID-19 and underlying diseases, the possibility of adverse effects of metformin like lactate acidosis should be taken into account.

## Data Availability Statement

The original contributions presented in the study are included in the article/supplementary material. Further inquiries can be directed to the corresponding authors.

## Author Contributions

MZ, MJN, MM: designed the study. MZ, SAN, MMZ: performed the search, study selection, and data synthesis. MJN, MZ, BH, SAN, MMZ: wrote the first draft of the manuscript. MJN, MM: revised the article. All authors contributed to the article and approved the submitted version.

## Conflict of Interest

The authors declare that the research was conducted in the absence of any commercial or financial relationships that could be construed as a potential conflict of interest.
